# Prolonged Bat Call Exposure Induces a Broad Transcriptional Response in the Male Fall Armyworm (*Spodoptera frugiperda*; Lepidoptera: Noctuidae) Brain

**DOI:** 10.3389/fnbeh.2019.00036

**Published:** 2019-02-26

**Authors:** Scott D. Cinel, Steven J. Taylor

**Affiliations:** ^1^Illinois Natural History Survey, Prairie Research Institute, University of Illinois at Urbana-Champaign, Champaign, IL, United States; ^2^Insect Evolution, Behavior, and Genomics Lab, Florida Museum of Natural History, University of Florida, Gainesville, FL, United States; ^3^Colorado College, Colorado Springs, CO, United States

**Keywords:** bat, moth, neurophysiology, stress, predation, *Spodoptera frugiperda*, transcriptomics, ultrasound

## Abstract

Predation risk induces broad behavioral and physiological responses that have traditionally been considered acute and transitory. However, prolonged or frequent exposure to predators and the sensory cues of their presence they broadcast to the environment impact long-term prey physiology and demographics. Though several studies have assessed acute and chronic stress responses in varied taxa, these attempts have often involved *a priori* expectations of the molecular pathways involved in physiological responses, such as glucocorticoid pathways and neurohormone production in vertebrates. While relatively little is known about physiological and molecular predator-induced stress in insects, many dramatic insect defensive behaviors have evolved to combat selection by predators. For instance, several moth families, such as Noctuidae, include members equipped with tympanic organs that allow the perception of ultrasonic bat calls and facilitate predation avoidance by eliciting evasive aerial flight maneuvers. In this study, we exposed adult male fall armyworm (*Spodoptera frugiperda*) moths to recorded ultrasonic bat foraging and attack calls for a prolonged period and constructed a *de novo* transcriptome based on brain tissue from predator cue-exposed relative to control moths kept in silence. Differential expression analysis revealed that 290 transcripts were highly up- or down-regulated among treatment tissues, with many annotating to noteworthy proteins, including a heat shock protein and an antioxidant enzyme involved in cellular stress. Though nearly 50% of differentially expressed transcripts were unannotated, those that were are implied in a broad range of cellular functions within the insect brain, including neurotransmitter metabolism, ionotropic receptor expression, mitochondrial metabolism, heat shock protein activity, antioxidant enzyme activity, actin cytoskeleton dynamics, chromatin binding, methylation, axonal guidance, cilia development, and several signaling pathways. The five most significantly overrepresented Gene Ontology terms included chromatin binding, macromolecular complex binding, glutamate synthase activity, glutamate metabolic process, and glutamate biosynthetic process. As a first assessment of transcriptional responses to ecologically relevant auditory predator cues in the brain of moth prey, this study lays the foundation for examining the influence of these differentially expressed transcripts on insect behavior, physiology, and life history within the framework of predation risk, as observed in ultrasound-sensitive Lepidoptera and other ‘eared’ insects.

## Introduction

Predator-induced stress has long fascinated biologists for its integrated, scalable effects on prey physiology, behavior (Slos and Stoks, 2008), and even spatiotemporal population demographics (Clinchy et al., 2013). Though a mechanistic understanding of the physiological responses that are induced by predation related stress in vertebrates has been known for some time, researchers interested in similar responses in invertebrate taxa, such as insects, now seek a similar descriptive model. The study of invertebrate stress responses has a rich history, yet the diversity of molecular components induced by various stressors has thus far stymied most attempts at holistic understanding. Recently, however, Adamo (2010, 2017a) demonstrated that the early stages of stress responses in insects are homologous, and likely anciently related, to vertebrate neurotransmitter signaling and downstream neurohormonal activation. The challenge remains, then, in describing the varied taxon- and tissue-specific responses seen in insects and elucidating the mechanisms responsible for inducing them.

Often before a predator has even localized its prey, a suite of adaptive behavioral and physiological responses which improve the chances of survival (Endler, 1991) are induced in prey organisms which may be eavesdropping on mechanical, auditory, visual, and chemosensory predation cues (Adamo et al., 2013). For instance, moths and butterflies that are sensitive to ultrasound display startle responses when exposed to synthetic broad frequency ultrasound (Roeder, 1966; Ratcliffe et al., 2008, 2011; ter Hofstede et al., 2011) and recorded bat calls (Acharya and McNeil, 1998; Rydell et al., 2003; Ratcliffe and Fullard, 2005), such as changing the course of flight, ceasing flight, accelerating, performing evasive flight maneuvers (Yack, 2004; Yack et al., 2007; Pfuhl et al., 2015), and/or calling back with jamming ultrasound themselves (Corcoran et al., 2009). Upon exposure to ultrasound, non-flying noctuid moths cease movement while many aerial noctuids exhibit evasive flight maneuvers, such as erratic changes in direction, loops, increases in flight velocity, and even falling to the ground (Surlykke and Miller, 1982). Moreover, when exposed to bat calls, many female and male tympanate moths alter their mating behavior by stopping pheromone release or ceasing flight, respectively (Acharya and McNeil, 1998). These behavioral responses, especially when borne out for an extended period of time, may contribute to patterns of stressor-induced gene regulation in insects that may contribute to reports of moths that display modified fecundity and life history patterns following prolonged exposure to recorded and synthetic bat ultrasound in a laboratory setting (Huang et al., 2003; Zha et al., 2008, 2013). For instance, *Plodia interpunctella* (Lepidoptera: Pyralidae) exposed to short bursts of ultrasound near their hearing range (approximately 50 kHz) respond by modifying mating behavior (Trematerra and Pavan, 1995) and long-term exposure even affects spermatophore quality and larval numbers by up to 75% (Kirkpatrick and Harein, 1965; Huang et al., 2003) while simultaneously reducing F_1_ larval weight and growth rates (Huang et al., 2003; Huang and Subramanyam, 2004). Conversely, long-term exposure to broadband ultrasound in *Helicoverpa armigera* (Lepidoptera: Noctuidae) significantly increased whole-body acetylcholinesterase activity (Zha et al., 2008), the number of spermatophores per female, and the number of eggs laid (Zha et al., 2013).

In order to maintain internal homeostasis during stressful periods, whether osmotically, metabolically, or otherwise, insects and most other forms of life evolved biomolecular signaling cascades, both intra- and extra-cellularly, that often regulate the expression of stress-related genes (Pauwels et al., 2005; Aruda et al., 2011; Yamaguchi et al., 2012; Roszkowski et al., 2016) and resulting behaviors, including vigilance (Lima, 1990; Kight and Swaddle, 2011) and modified activity patterns (Abramsky et al., 2014). Further, these responses mediate cellular metabolism and the degradative effects of prolonged and persistent stressor exposure, including oxidative damage (Slos and Stoks, 2008; Clinchy et al., 2013), protein misfolding (Fleshner et al., 2004; Even et al., 2012), and organelle turnover (Salvetti et al., 2000; Gesi et al., 2002). However, individual cells can respond to stressful conditions by activating transcriptional pathways that usually produce one or more damage-mitigating antioxidant enzymes or protein folding chaperones, such as the heat shock proteins (Hsps). Though these molecular defenses promote physiological homeostasis in the short-term, prolonged periods of stress clearly influence the life history and fitness of many species. Even though biologists have long recognized the importance of stress hormone signaling for initiating behavioral and physiological defenses to predation, the cellular- and tissue-level mechanisms by which long-term acclimation to predation risk can influence the life history and fitness of prey species remains unclear, particularly among insects.

In this study, we exposed adult male fall armyworm moths to recorded ultrasonic foraging and attack calls of three insectivorous bat species over an 8-h period to test the influence of an ecologically relevant auditory cue of predation on the cellular physiology of the noctuid brain. The fall armyworm, though a non-model species itself, is in the same family as the corn earworm (*Helicoverpa zea*), whose annotated reference genome was recently published (Pearce et al., 2017) and whose old world sister species, *H. armigera*, has long been a prominent subject in insect auditory neuroethology studies for its dramatic neurobehavioral responses to ultrasound. The fall armyworm, and many other tympanic moths, thus make prime candidates for describing the biochemical and cellular responses that have evolved to cope with prolonged predation risk in insects. We hypothesized that a broader transcriptomic response would be induced in the brains of cue-exposed relative to unexposed individuals. Further, we predicted this response might involve transcripts pertaining to the following physiological functions: (1) intracellular secondary messenger systems, (2) antioxidant and Hsp activity, and (3) gene regulation.

## Materials and Methods

Fall armyworm larvae were purchased from Frontier Agricultural Sciences (Newark, DE, United States) under USDA APHIS PPQ 526 permit (P526P-04080) and were shipped over-night as second and third instar larvae. Upon arrival at the Illinois Natural History Survey, Prairie Research Institute, University of Illinois at Urbana-Champaign in Champaign, IL, United States, larvae were transferred to individual 59 mL (2 oz.) plastic cups filled with 10–15 mL of standard lepidopteran diet and reared in an environmental chamber (Percival Scientific, Perry, IA, United States) at 30 ± 1°C and 75 ± 5% RH, with a photoperiod of 16 h light/8 h dark. Larvae fed *ad libitum* on a modified standard larval lepidopteran diet (Sims, 1998; Cohen, 2001; Elvira et al., 2010) prepared every 2 weeks. This diet consisted of 13 g agar, 770 mL distilled water, 31.5 g vitamin-free casein, 24 g sucrose, 27 g wheatgerm, 9 g Wesson’s salt mix, 10 g alphacel, 5 mL 4 M potassium hydroxide, 18 g Vanderzant’s vitamins, 1.6 g sorbic acid, 1.6 g methyl paraben, 3.2 g ascorbic acid, 0.12 g streptomycin salt, 4 mL wheatgerm oil, and 2 mL 10% formaldehyde. We blended the casein, sucrose, wheatgerm, Wesson’s salt mix, alphacel, 220 mL distilled water, and potassium hydroxide on high for 5 min, to which we added 550 mL of mildly boiling distilled, deionized water mixed with agar. We then blended the mixture for another 5 min and allowed it to cool to 60°C before we added Vanderzant’s vitamins, sorbic acid, methyl paraben, ascorbic acid, streptomycin, wheatgerm oil, and formaldehyde and blended for a final 5 min. We poured 10–15 mL of the cooled diet into each 2 oz. rearing cup and allowed them to solidify in a cold-room for at least 30 min.

We then placed a larva into each filled cup and secured a lid in which two holes had been punched using a No. 1 insect pin. Once a larva cleared its gut before pupation, we transferred it to a shallow Tupperware container (29.4 cm × 15.1 cm × 10.5 cm) filled with 3.5 cm of loose potting soil (SunGro Horticulture, Vancouver, BC, Canada). Once per day, this soil was sifted gently by hand to extract any pupae, which were placed in a separate 30.48 cm^3^ mesh cage (BioQuip Products, Inc., Compton, CA, United States) with a mesh-size of 51.15 holes/cm^2^ within the environmental chamber until emergence.

Upon emergence, adults were transferred to a similar mesh cage and allowed to mate. Twice daily, we saturated the sides of the mesh cage with a 10% sucrose solution to allow feeding. To avoid the possible confounding effects of shipment and the change in diet undergone by the generation of larvae received from Frontier Agricultural Sciences, F_1_ eggs were collected daily from within this cage and placed in small plastic containers within the rearing chamber. Once hatched, we reared F_1_ larvae as above until emergence as adults.

### Predator Cue Exposure

A random sample of four control and four experimental F_1_ adult males (sex determined by visual inspection of terminal pupal abdominal segment) were selected for use in trials 24–48 h post-eclosion. Females were not used, as female noctuid moths broadcasting pheromones are often sedentary (Stelinski et al., 2014) and may be preyed upon less frequently by aerial-hawking insectivorous bats. Three individual recordings were sampled at 480 kHz, 16-bit format and concatenated with 10 s of silence between each call. The calls consisted of (1) a 4.27 s *Molossus molossus* (Chiroptera: Molossidae) attack call, (2) a 1.51 s *Myotis nigricans* (Chiroptera: Vespertilionidae) foraging call, and (3) a 2.92 *Saccopteryx bilineata* (Chiroptera: Emballonuridae) foraging call. These three neotropical bat species were selected specifically because the neurophysiological response of *S. frugiperda* auditory neurons to these species’ calls have been explicitly described (Mora et al., 2014), they each represent a ubiquitous species throughout much of *S. frugiperda*’s range in the Americas (Mora et al., 2004; Jung et al., 2007; Surlykke and Kalko, 2008), and they likely represent novel predators for the lab-reared, United States-based *S. frugiperda* colony used in this study. Further, these species produce calls of varying amplitudes and frequencies that together span the known response curve of the *S. frugiperda* tympanum (Mora et al., 2004, 2014). Specifically, *M. molossus*, *M. nigricans*, and *S. bilineata* broadcast at 20–50 (Mora et al., 2004), 50–85, and 45–55 (Jung et al., 2007) kHz, respectively, whereas *S. frugiperda* responds optimally to sounds within 20–50 kHz (Mora et al., 2014). The individual sound files were processed in Audacity v. 2.1.0. to reduce background ultrasound by applying a 20-dB noise reduction filter to frequencies lower than 30 kHz with moderate sensitivity (10.0) and re-sampled each file at 195.3125 kHz to meet the limitations of our playback system. This down-sampling attenuated frequencies greater than 75 kHz (Tucker-Davis Technologies, personal communication), but reproduced the bat calls faithfully within the 20–50 kHz optimal hearing range reported for noctuid moths (Fullard, 1988; Norman and Jones, 2000). The resulting 38 s file was then broadcast on a loop for the 8-h duration of each experimental trial while control trials consisted of an identical setup with no sound played whatsoever. Calls were broadcast via a Tucker-Davis Technologies (TDT; Alachua, FL, United States) System 3 amplifier powering an ES1 electrostatic free-field speaker (TDT) that was situated 30 cm from the center of the cage in a soundproof, anechoic chamber at the Beckman Institute, University of Illinois at Urbana-Champaign in Urbana, IL, United States. The RPvdsEx software suite v. 80 (TDT) was used to process and playback the audio file via the TDT RP2.1 processor, ED1 Electrostatic Speaker Driver, and SA1 Stereo Amplifier tandem setup. Each of the four, 8-h replicate exposure and control trials took place on alternating nights in September 2017 from 22:00 to 05:00.

### Sample Preparation and Sequencing

Post-exposure, each moth was placed into a 2 mL vial and immediately immersed in liquid nitrogen. After 30 s, the moth was removed from the vial and transferred quickly to a Petri dish on dry ice. After the head was removed, we immersed it in RNAlater stabilization solution (Life Technologies). Upon immersion, scales on the head capsule were removed by scraping with scalpel, and a 1 mm × 1 mm section of cuticle was cut to expose the brain tissue directly to RNAlater. We then dissected the brain from the head capsule, rinsed it with fresh RNAlater solution, placed it in a 2 mL microtube of fresh RNAlater solution, and stored it at 2°C until all samples had been collected.

RNA was extracted from each brain using a PicoPure RNA Isolation Kit (Arcturus Bioscience). RNA was eluted in 30 μL of RNase-free water and stored at -80°C until further analysis. Before freezing, 3.5 μL aliquots were removed from each extract and used for RNA quantification via a NanoDrop (Thermo Fisher Scientific) spectrophotometer and a Qubit fluorometer (Life Technologies) using a Qubit RNA HS Assay Kit (Life Technologies). After a 1:10 or 1:15 dilution based on each sample’s concentration, we submitted these subsamples to the Functional Genomics Unit of the University of Illinois at Urbana-Champaign’s (UIUC) Roy J. Carver Biotechnology Center to confirm RNA quality with a Bioanalyzer RNA 6000 Pico chip (Agilent).

We then submitted each RNA extract to the UIUC Roy J. Carver Biotechnology Center’s High-Throughput Sequencing and Genotyping Unit for library preparation and sequencing. Strand-specific cDNA libraries were prepared using an Illumina TruSeq Stranded mRNA Sample Prep Kit (dUTP based) according to manufacturer specifications and quantified by quantitative polymerase chain reaction (qPCR). The eight samples were multiplexed on a single lane of an Illumina 2500 sequencer and the RNA fragments were sequenced using Illumina’s HiSeq SBS Sequencing Kit v4 for 101 cycles with a 100 nt paired-end read length.

### Raw mRNA Read Preprocessing

Sequence files were demultiplexed with Illumina’s bcl2fstq v. 217.1.14 conversion software. To ascertain raw read quality, we used FastQC v. 0.11.2 (Andrews, 2010) with default settings on each set of reads. We then preprocessed the raw reads by performing adapter trimming, quality filtering, and *in silico* normalization. Adapter trimming and quality filtering was achieved using Trimmomatic v. 0.33 (Bolger et al., 2014) in palindrome mode to search for and remove adapter sequences and low quality bases. To remove redundant reads and improve transcriptome assembly performance, the remaining reads were then digitally normalized to a coverage depth of 50× via the Trinity transcriptome assembly suite v. 2.1.1 (Grabherr et al., 2011; Haas et al., 2013).

### *De novo* Transcriptome Assembly, Annotation, and Quality Assessment

To our knowledge, there is no publicly available annotated reference genome for *Spodoptera frugiperda*; therefore, we chose to build a *de novo* transcriptome assembly with the pre-processed reads using the Trinity assembler v. 2.1.1 (Grabherr et al., 2011). We designated the sequence-specific strand orientation to ‘reverse-forward’ (RF) when possible. The quality of the resulting transcriptome was then assessed using TransRate v. 1.0.1 (Smith-Unna et al., 2016) and BUSCO v. 3 (Simão et al., 2015). We then utilized the Annocript v. 2.0 automated transcriptome annotation algorithm (Musacchia et al., 2015) to complete sequence-similarity searches on each assembled transcript against the National Center for Biotechnology Information (NCBI)’s non-redundant nucleotide database using BLAST+ v. 2.2.30 (Camacho et al., 2009). We selected the UniRef90 protein database (Boutet et al., 2016) to screen for computationally derived protein annotations. Annocript first downloaded the UniRef90 database, stored it in a MySQL v. 7.3 (Oracle Corporation, Redwood City, CA, United States) database, and indexed it for faster searches (Camacho et al., 2009). Annocript carried out BLASTX searches against the UniRef90 database and reported those hits with an e-value < 1e-5. Annocript output a tab-delimited feature map file containing the collated annotation information for each putative assembled transcript.

### Read Alignment, Abundance, and Differential Expression Analysis

Following annotation, we indexed the transcriptome in Kallisto (Bray et al., 2016) using the ‘kallisto index’ command before aligning each sample’s reads against the index using the ‘kallisto quant’ command to select 250 bootstrap replicates each. In R v. 3.5.1 (R Core Team, 2014), we utilized the packages ‘edgeR’ v. 3.12.1 (Robinson et al., 2009) and ‘limma’ v. 3.26.9 (Ritchie et al., 2015) to import the estimated read counts and perform DE statistical analyses. First, we used the trimmed mean of M-values (TMM) normalization method (Robinson and Oshlack, 2010) to account for small biases in each sample’s overall read library size. To filter out transcripts with low or no expression estimates in one or more grouped replicates (Rau et al., 2013), we calculated the counts per million (CPM) mapped reads for each transcript and removed those with a CPM < 1.

**FIGURE 1:**
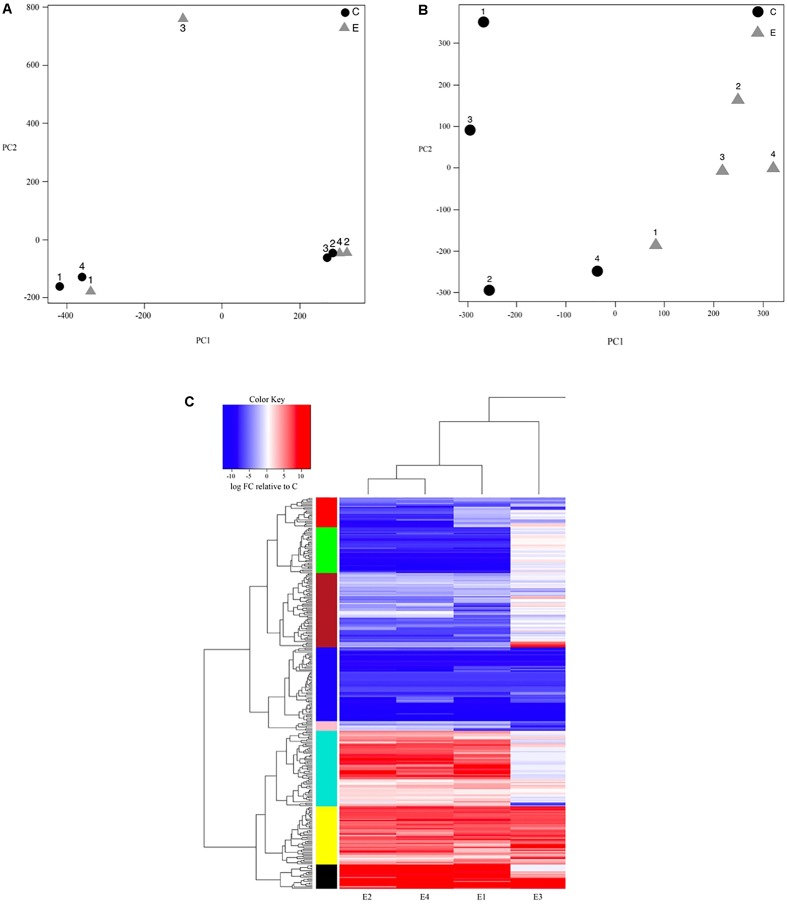
**(A)** Principal components plot showing sample clustering based on the first two principal components of variation in log-based counts per million read estimates for both control (C; black circle) and bat-ultrasound exposed (E; gray triangle) *Spodoptera frugiperda* moths; numbers (1–4) represent replicate samples from each of the control and exposure groups. **(B)** Principal components plot after surrogate variable analysis was performed to account for unexpected batch effects showing sample clustering based on the first two principal components of variation in log-based counts per million read estimates for both control (C; black circle) and bat-ultrasound exposed (E; gray triangle) adult male *Spodoptera frugiperda* moths; numbers (1–4) represent replicate samples from each of the control and exposure groups. **(C)** Transcript expression heatmap detailing the up- (red) and down- (blue) regulation (log_2_FC) of each transcript relative to the mean expression of the control group across bat-ultrasound exposed (E) adult male *Spodoptera frugiperda* moths; samples (horizontal axis) and transcripts (vertical axis) are clustered according to expression similarity (stacked multicolored bars).

We then visually assessed the presence of batch effects in our data by performing principal components analysis (PCA) on log-transformed CPM expression values across each sample using the ‘affycoretools’ v. 1.42.0 (MacDonald, 2008) package in R. To account for a large amount of expression variation observed between replicate samples (Figure 1A), we used the ‘sva’ v. 3.18.0 (Leek et al., 2012, 2010) package to explicitly model three identified surrogate variables as covariates. After adding these covariates to our dataset, we log-transformed all CPM estimates to prepare for linear modeling. We then used the ‘limma’ package and its ‘voom’ function (Law et al., 2014) to fit a negative binomial linear model and proceeded to compute pairwise t-statistics, F-statistics, and log-odds of differential expression for each transcript according to exposure type using empirical Bayes (Smyth, 2004). The resulting differentially expressed transcripts were filtered by selecting only those with false discovery rate (FDR)-adjusted *p*-values < 0.05 and a fold-change > 2 to account for multiple testing bias on *p*-value significance (Benjamini and Hochberg, 1995; Benjamini and Heller, 2007).

To produce a heatmap of gene expression across the samples, we scaled each transcript’s associated fold-change to the mean fold-change observed in all transcripts from the control group. To assess similarity in expression between samples, we used a hierarchical clustering method based on a distance matrix compiled by taking the maximal distance between any two expression values in each sample via the ‘fastcluster’ package v. 1.1.20 (Müllner, 2013) in R. The resultant base dendrogram of similarity between individual transcripts was then used to identify the most appropriate level at which to cluster our transcripts using the R package ‘dynamicTreeCut’ v. 1.63-1 (Langfelder et al., 2008). We chose to use the ‘hybrid’ method to first identify large, base clusters following four criteria: (1) each cluster must contain ≥2 transcripts; (2) transcripts that are too distant from a cluster are excluded, even if they occur on the same branch; (3) each preliminary cluster must be distinct from those clusters near to it; and (4) the tips of each preliminary cluster must be tightly connected. Once these clusters were identified, any transcripts not previously assigned were placed in the closest neighboring cluster. Using ‘cdbfasta’ v. 0.99^1^, we then retrieved the sequences and Gene Ontology (GO) terms associated with these differentially expressed (DE) transcripts from our annotated transcriptome for downstream functional GO enrichment analysis. Figures were constructed using the R packages ‘graphics’ v. 3.2.4, ‘grDevices’ v. 3.2.4, ‘rgl’ v. 0.95.1441, and ‘gplots’ v. 2.17.0.

### Functional Gene Ontology Term Enrichment and KEGG Pathway Analyses

To obtain a broader perspective on the function of our DE transcripts and how they may be related, we tested their associated annotated GO terms for statistically significant over- and under-representation via GO term enrichment analysis. The background set of transcripts we used to test our DE set against included all GO annotations from base transcriptome. Using the ‘Biological Networks Gene Ontology’ (BiNGO) plugin v. 3.0.3 (Maere et al., 2005) in the Cytoscape platform v. 3.3.0 (Shannon et al., 2003), we tested for both over- and under-representation using a hypergeometric test at an FDR-adjusted *p*-value < 0.05. Each differentially expressed transcript was also annotated using the automated BlastKOALA (Kanehisa et al., 2016) KEGG pathway webserver and analyzed manually for functional relevance.

### Data Availability

The raw sequence reads have been uploaded to the NCBI Sequence Read Archive (SRA) database (accessions: SRR3406020, SRR3406031, SRR3406036, SRR3406052, SRR3406053, SRR3406054, SRR3406055, SRR3406059) and are also available through the BioProject accession PRJNA318819^2^. The transcriptome has been archived to NCBI’s Transcriptome Shotgun Assembly database under accession GESP00000000; the version used here is GESP00000000.1. A repository containing R scripts and output files from all analyses downstream of assembly is also hosted on GitHub^3^.

## Results

### RNA Extraction, Library Preparation, and Read Processing

Each RNA extract was found to produce satisfactory yields, and these were subsequently used in downstream analyses. Total RNA concentration in each sample was generally consistent between NanoDrop and Qubit estimates, the absorbance ratios signified little if any contamination (A260/280 > 2) and the bioanalyzer assay revealed each sample consisted of high-quality RNA with negligible signs of degradation (RIN > 8; Table 1). Our cDNA fragment lengths after library preparation ranged from 80 to 700 bp, with an average of 300 bp. Each sample produced similar numbers of reads, ranging between 28.3 million and 31.1 million. The average quality scores for each base in each sample were ≥33 (phred-33 scaling), allowing us to proceed without sequencing error correction. Preprocessing steps led to less than 0.12% of reads being removed in each sample, and the GC content of the samples ranged from 42 to 45% post-trimming.

**Table 1:** Total RNA concentration, absorbance values, absorbance ratios, and RNA Integrity Number (RIN) for each brain tissue RNA extraction from control (C) and bat ultrasound-exposed (E) adult male *Spodoptera frugiperda* moths.

Sample ID	NanoDrop concentration (ng/μL)	Qubit concentration (ng/μL)	A260	A280	A260/280	RIN
C1	49.65	49.7	1.241	0.593	2.09	8.3
C2	54.78	58.4	1.370	0.636	2.15	8.5
C3	42.76	44.2	1.069	0.491	2.18	8.7
C4	46.21	49.1	1.155	0.539	2.14	8.3
E1	74.34	71.8	1.859	0.869	2.14	8.7
E2	52.91	56.6	1.323	0.638	2.07	8.8
E3	66.32	61.9	1.658	0.768	2.16	8.7
E4	35.29	38.1	0.882	0.419	2.11	9.0


### *De novo* Transcriptome Assembly Statistics

Our transcriptome contained a total of 27,734 putative transcript contigs in total, ranging in length from 124 to 38,522 bp with an average of 1,399.9 bp. The contig N50 of our assembly was 2,933 bp and 40.9% of the contigs exceeded 1,000 bp in length. Out of 303 eukaryotic orthologs used as a reference in BUSCO, we identified 290 (95.7%) complete matches, with 234 single-copy and 56 duplicate hits, along with three fragmented and ten missing orthologs. Annocript annotated 10,367 (37.38%) contigs with reliable protein annotations from significant (e-value < 10^-5^) BLASTX hits using the UniRef90 database. Of these hits, 97.0% and 93.6% were annotated to species of Insecta and Lepidoptera, respectively. Mapping GO annotations to these hits resulted in 6,476 GO annotations present in the transcriptome, with 4,075 gene products attributed to biological processes, 815 to cellular components, and 1,586 to molecular function. The top GO terms attributed to the largest numbers of transcript contigs included ‘integral to membrane’ (GO:0016021), ‘nucleic acid binding’ (GO:0003676), ‘ATP binding’ (GO:0005524), ‘nucleus’ (GO:0005634), and ‘zinc ion binding’ (GO:0008270).

### Read Alignment and Abundance Quantification

On average, 36.59% ± 0.573% (95% CI) of reads from each sample mapped to the transcriptome. TMM normalization resulted in normalization factors ranging from 0.915 to 1.104, which we then multiplied by our actual library sizes to find our final effective library sizes. After filtering low and no expression transcripts with <1 CPM, 17,558 out of 27,734 (63.3%) were retained for DE analysis.

### Differential Transcript Expression Analysis

Our initial PCA indicated strong, unexpected clustering of samples along the first two principal axes (Figure 1A), leading us to use surrogate variable analysis in effort to remove potential unaccounted batch effects. We found three significant surrogate variables that we included in our negative binomial regression model as covariates, resulting in clear clustering of samples by experimental group (Figure 1B). Further, we improved our detection of significant DE transcripts at a FDR < 0.05 with ≥2-fold change in expression from 75 to 290 transcripts after including the covariates (Figure 1C). Of the 290 DE transcripts, 146 (50.3%) had significant BLASTX hits (e-value < 1e-5), though 44 (15.2%) had uncharacterized functions (Tables 2, 3). The top 11 organisms with the highest number of hits to DE transcripts were all also lepidopteran taxa, with most pertaining to *Amyelois transitella* (Lepidoptera: Pyralidae). Of the 290 DE transcripts, 117 were upregulated while 173 were downregulated.

**Table 2:** List of differentially upregulated (log_2_-transformed fold change) transcripts recovered from brain tissue mRNA extractions in bat call-exposed *Spodoptera frugiperda* adult male moths relative to controls, including the most significant (e-value < 1e-5) BLASTX protein annotation from the UniRef90 database and the organism from which the annotation is derived.

Transcript ID	Log_2_ fold change	Ave. expression	*P*-value	FDR-adjusted *P*-value	Top UniRef90 BLASTX Hit	E-value	Organism
TRINITY_DN2230_c0_g1_i1	11.4500	-1.4769	0.0000	0.0000	-	-	-
TRINITY_DN37212_c0_g1_i3	9.8601	-0.7439	0.0000	0.0000	X-linked retinitis pigmentosa GTPase regulator homolog	0	*Bombyx mori*
TRINITY_DN36403_c0_g1_i3	9.6654	-2.1606	0.0000	0.0000	Calcium uniporter protein mitochondrial	0	*Papilio polytes*
TRINITY_DN30280_c6_g1_i3	9.6383	-1.3637	0.0000	0.0000	-	-	-
TRINITY_DN38404_c0_g2_i3	9.5504	-1.0808	0.0000	0.0000	-	-	-
TRINITY_DN33318_c7_g1_i1	9.4738	-2.2233	0.0000	0.0000	-	-	-
TRINITY_DN33671_c2_g1_i8	9.3876	-1.2532	0.0000	0.0000	-	-	-
TRINITY_DN35646_c2_g4_i3	9.0219	-1.4826	0.0003	0.0594	Uncharacterized protein LOC105386011	1.00E-43	*Plutella xylostella*
TRINITY_DN37234_c0_g1_i10	8.6926	-2.5344	0.0000	0.0000	-	-	-
TRINITY_DN30400_c2_g1_i4	8.6099	-2.5413	0.0000	0.0000	-	-	-
TRINITY_DN38739_c1_g1_i16	8.5436	-1.2902	0.0000	0.0000	Protein polybromo-1	0	*Papilio* sp.
TRINITY_DN35646_c2_g4_i5	8.4296	-2.0614	0.0000	0.0130	Uncharacterized protein LOC105386011	4.00E-42	*Plutella xylostella*
TRINITY_DN34405_c8_g8_i2	8.4089	-2.3193	0.0000	0.0000	-	-	-
TRINITY_DN40154_c8_g1_i2	8.2644	-1.0302	0.0000	0.0030	-	-	-
TRINITY_DN37042_c2_g2_i1	8.1724	-2.2874	0.0000	0.0006	-	-	-
TRINITY_DN40225_c4_g3_i1	7.9335	-2.4214	0.0000	0.0000	-	-	-
TRINITY_DN37134_c1_g1_i13	7.7773	-0.8312	0.0000	0.0111	-	-	-
TRINITY_DN34896_c2_g1_i1	7.7609	-2.3763	0.0000	0.0017	-	-	-
TRINITY_DN37497_c1_g1_i16	7.7348	-2.7427	0.0000	0.0024	Nuclear factor 1 C-type-like	0	*Plutella xylostella*
TRINITY_DN33065_c0_g2_i1	7.7268	-2.6286	0.0000	0.0008	Aminoacylase-1-like	1.00E-92	*Amyelois transitella*
TRINITY_DN32642_c3_g3_i12	7.6793	-2.1043	0.0000	0.0000	Regulatory-associated protein of TOR	0	*Bombyx mori*
TRINITY_DN38729_c4_g1_i3	7.3105	-0.6533	0.0002	0.0484	Phosphatidate cytidylyltransferase	0	*Ditrysia* sp.
TRINITY_DN36166_c2_g1_i1	7.1246	-2.2272	0.0000	0.0000	-	-	-
TRINITY_DN29778_c0_g1_i3	7.1124	-2.3090	0.0000	0.0077	-	-	-
TRINITY_DN31620_c0_g1_i6	7.0401	-3.2045	0.0000	0.0007	Uncharacterized protein	0	*Papilio* sp.
TRINITY_DN34936_c0_g1_i3	6.9579	-2.4491	0.0000	0.0010	Myoneurin-like	1.00E-76	*Bombyx mori*
TRINITY_DN37234_c0_g1_i8	6.8524	-2.5641	0.0000	0.0012	-	-	-
TRINITY_DN29467_c1_g1_i4	6.8383	-2.4531	0.0000	0.0000	-	-	-
TRINITY_DN25058_c0_g1_i3	6.8317	-3.0539	0.0000	0.0000	Putative ecdysone oxidase	2.00E-15	*Operophtera brumata*
TRINITY_DN25058_c0_g1_i2	6.7810	-3.2099	0.0000	0.0000	Mitochondrial choline dehydrogenase	3.00E-21	*Operophtera brumata*
TRINITY_DN36104_c1_g1_i1	6.7539	-1.9239	0.0000	0.0037	Pro-resilin-like	2.00E-17	*Amyelois transitella*
TRINITY_DN37496_c0_g1_i3	6.7062	-2.3284	0.0000	0.0000	-	-	-
TRINITY_DN36563_c0_g1_i5	6.6760	-2.0072	0.0000	0.0149	Axin	0	*Papilio* sp.
TRINITY_DN37270_c2_g1_i4	6.6685	-2.6072	0.0001	0.0178	-	-	-
TRINITY_DN39071_c0_g1_i2	6.6680	-3.4169	0.0000	0.0088	Putative uncharacterized protein	0	*Tribolium castaneum*
TRINITY_DN39461_c2_g2_i3	6.5369	-3.3048	0.0000	0.0000	-	-	-
TRINITY_DN39385_c2_g1_i1	6.4561	0.4404	0.0000	0.0007	-	-	-
TRINITY_DN36280_c3_g1_i10	6.4352	-1.6074	0.0001	0.0339	Putative uncharacterized protein	3.00E-08	*Culex quinquefasciatus*
TRINITY_DN37496_c0_g1_i4	6.4322	-2.7123	0.0000	0.0000	-	-	-
TRINITY_DN37496_c0_g1_i6	6.3548	-2.6646	0.0000	0.0000	-	-	-
TRINITY_DN37496_c0_g2_i12	6.3394	-2.4473	0.0000	0.0000	Uncharacterized protein	2.00E-96	*Danaus plexippus*
TRINITY_DN37877_c0_g1_i2	6.3218	-3.3586	0.0000	0.0004	-	-	-
TRINITY_DN30964_c1_g2_i5	6.3073	-2.7133	0.0000	0.0002	Ester hydrolase C11orf54 homolog	3.00E-136	*Amyelois transitella*
TRINITY_DN34741_c4_g2_i4	6.2842	-3.2922	0.0001	0.0191	-	-	-
TRINITY_DN37496_c0_g1_i10	6.2575	-2.7025	0.0000	0.0000	-	-	-
TRINITY_DN40271_c4_g1_i5	6.2438	-3.0788	0.0000	0.0004	-	-	-
TRINITY_DN38404_c0_g2_i2	6.2234	-2.7265	0.0000	0.0000	Acyl-CoA synthetase short-chain family member 3 mitochondrial	0	*Amyelois transitella*
TRINITY_DN32565_c0_g2_i3	6.2016	-2.6574	0.0000	0.0000	-	-	-
TRINITY_DN39907_c0_g1_i3	6.1037	-2.8112	0.0001	0.0207	Coronin-6 isoform X1	0	*Obtectomera* sp.
TRINITY_DN37997_c0_g1_i2	6.0595	-2.4577	0.0000	0.0001	Type II inositol 1,4,5-trisphosphate 5-phosphatase	0	*Papilio* sp.
TRINITY_DN31620_c0_g1_i1	6.0511	-3.5720	0.0000	0.0002	Uncharacterized protein	0	*Papilio* sp.
TRINITY_DN37364_c0_g5_i1	5.9831	-3.5345	0.0000	0.0000	Cystinosin homolog isoform X1	3.00E-12	*Plutella xylostella*
TRINITY_DN38655_c0_g1_i1	5.9808	-3.0258	0.0000	0.0078	ATP-binding cassette sub-family G member 5	0	*Bombyx mori*
TRINITY_DN32711_c0_g1_i3	5.9679	-2.9031	0.0000	0.0007	Doublesex- and mab-3-related transcription factor 3	6.00E-134	*Amyelois transitella*
TRINITY_DN29332_c0_g1_i3	5.8838	-3.1589	0.0000	0.0027	-	-	-
TRINITY_DN35731_c2_g1_i1	5.8568	-1.1235	0.0002	0.0488	-	-	-
TRINITY_DN39575_c4_g3_i5	5.8164	-3.7350	0.0001	0.0360	-	-	-
TRINITY_DN33671_c2_g1_i7	5.7500	-3.1041	0.0000	0.0000	-	-	-
TRINITY_DN27959_c1_g1_i1	5.7076	-3.3723	0.0000	0.0004	Uncharacterized protein	7.00E-98	*Bombyx mori*
TRINITY_DN30778_c0_g1_i4	5.6997	-2.5512	0.0001	0.0233	Putative chemosensory ionotropic receptor IR75d (Fragment)	0	*Spodoptera littoralis*
TRINITY_DN33595_c2_g1_i8	5.6920	-2.9806	0.0000	0.0001	Uncharacterized protein	0	*Bombyx mori*
TRINITY_DN37234_c0_g2_i1	5.6403	-2.9497	0.0000	0.0023	-	-	-
TRINITY_DN37848_c1_g3_i6	5.6040	-3.0244	0.0000	0.0000	Mutant cadherin	8.00E-16	*Helicoverpa armigera*
TRINITY_DN16945_c0_g1_i1	5.4814	-3.4197	0.0000	0.0016	-	-	-
TRINITY_DN37364_c0_g1_i1	5.4556	-3.7441	0.0000	0.0000	Heat shock protein 67B2-like isoform X2	1.52E-87	*Helicoverpa armigera*
TRINITY_DN38899_c0_g1_i1	5.2744	4.5685	0.0000	0.0016	Uncharacterized protein	8.00E-168	*Danaus plexippus*
TRINITY_DN38884_c1_g1_i5	5.1372	-3.8241	0.0000	0.0009	-	-	-
TRINITY_DN33012_c0_g1_i2	5.1079	-1.6502	0.0000	0.0134	-	-	-
TRINITY_DN37390_c4_g4_i3	5.0402	-3.2212	0.0001	0.0341	-	-	-
TRINITY_DN33831_c3_g1_i12	5.0258	-3.2425	0.0001	0.0177	-	-	-
TRINITY_DN38345_c0_g1_i5	4.9378	-3.2726	0.0000	0.0016	Dorsal 1a	5.00E-100	*Spodoptera litura*
TRINITY_DN40225_c4_g3_i3	4.8838	-2.1985	0.0000	0.0100	Glutathione *S*-transferase 2-like	1.19E-127	*Spodoptera litura*
TRINITY_DN36290_c2_g1_i3	4.8566	-3.2157	0.0000	0.0003	-	-	-
TRINITY_DN39385_c0_g1_i1	4.8501	1.4926	0.0000	0.0001	-	-	-
TRINITY_DN32186_c0_g1_i3	4.7616	-1.9394	0.0001	0.0286	-	-	-
TRINITY_DN37042_c3_g1_i2	4.6684	-2.2283	0.0000	0.0032	-	-	-
TRINITY_DN35392_c2_g1_i10	4.6570	-3.2271	0.0001	0.0308	Uncharacterized protein LOC107191251	8.00E-94	*Dufourea novaeangliae*
TRINITY_DN33705_c1_g1_i5	4.6475	-3.9626	0.0002	0.0392	Synaptic vesicle glycoprotein 2B-like	1.00E-112	*Amyelois transitella*
TRINITY_DN38135_c7_g3_i1	4.6201	-2.5497	0.0001	0.0266	-	-	-
TRINITY_DN33081_c0_g1_i4	4.6060	-2.4724	0.0001	0.0320	Dual specificity protein phosphatase 18	4.00E-28	*Operophtera brumata*
TRINITY_DN36290_c2_g1_i9	4.4223	-2.1098	0.0000	0.0061	Sodium/potassium-transporting ATPase subunit beta-2-like	6.00E-19	*Amyelois transitella*
TRINITY_DN38768_c0_g1_i1	4.3666	-0.2316	0.0000	0.0035	Uncharacterized protein LOC106125418	7.00E-147	*Papilio* sp.
TRINITY_DN40225_c4_g3_i4	4.3080	1.0204	0.0000	0.0024	-	-	-
TRINITY_DN35309_c0_g1_i1	4.2933	-1.4013	0.0003	0.0654	Uncharacterized protein LOC105383334	2.00E-52	*Plutella xylostella*
TRINITY_DN39696_c4_g6_i1	4.2733	-3.4922	0.0001	0.0201	-	-	-
TRINITY_DN39395_c1_g1_i5	4.1823	-3.4153	0.0000	0.0002	Serine/arginine repetitive matrix protein 1-like isoform X1	6.00E-135	*Papilio xuthus*
TRINITY_DN39527_c0_g1_i11	3.7373	6.2709	0.0000	0.0027	Z band alternatively spliced PDZ-motif protein 66	1.00E-41	*Papilio xuthus*
TRINITY_DN38817_c2_g2_i4	3.6350	-2.4653	0.0001	0.0233	Uncharacterized protein (Fragment)	1.00E-94	*Pararge aegeria*
TRINITY_DN38768_c0_g1_i3	3.4858	1.2063	0.0000	0.0023	Uncharacterized protein LOC106125418	3.00E-86	*Papilio* sp.
TRINITY_DN37183_c3_g1_i4	3.4076	3.6403	0.0001	0.0269	-	-	-
TRINITY_DN31771_c5_g1_i1	3.3905	-3.4420	0.0002	0.0363	-	-	-
TRINITY_DN36952_c0_g1_i7	3.3250	-1.6521	0.0001	0.0191	Cytochrome CYP341B3	0	*Spodoptera littoralis*
TRINITY_DN25843_c0_g2_i1	2.9305	-0.9304	0.0000	0.0134	-	-	-
TRINITY_DN39385_c1_g2_i2	2.8797	-1.0180	0.0001	0.0214	-	-	-
TRINITY_DN39461_c2_g2_i6	2.8066	0.9131	0.0000	0.0093	-	-	-
TRINITY_DN31963_c0_g1_i4	2.7588	-2.9390	0.0001	0.0238	-	-	-
TRINITY_DN36997_c1_g1_i5	2.6367	2.6586	0.0002	0.0402	-	-	-
TRINITY_DN39518_c1_g1_i3	2.6160	1.7318	0.0002	0.0431	ATP-binding cassette sub-family G member 8	0	*Amyelois transitella*
TRINITY_DN37039_c2_g2_i5	2.5625	0.4671	0.0002	0.0484	Phosphatidylglycero phosphatase and protein-tyrosine phosphatase 1	5.00E-123	*Amyelois transitella*
TRINITY_DN35489_c0_g1_i7	2.4620	2.2836	0.0000	0.0052	-	-	-
TRINITY_DN39905_c2_g3_i1	2.3712	-0.0139	0.0000	0.0077	-	-	-
TRINITY_DN35975_c0_g1_i7	2.3211	-0.8273	0.0000	0.0025	Protein Gawky	0	*Papilio* sp.
TRINITY_DN35944_c2_g2_i1	2.3075	-3.1464	0.0001	0.0290	Uncharacterized protein	3.00E-10	*Papilio xuthus*
TRINITY_DN40277_c8_g2_i4	2.0996	-0.4446	0.0001	0.0237	Uncharacterized protein LOC106713896 partial	2.00E-19	*Papilio machaon*
TRINITY_DN33887_c0_g2_i12	1.9515	-1.5605	0.0001	0.0237	Ubiquitin (fragment)	2.00E-57	*Protostomia* sp.
TRINITY_DN37783_c3_g1_i2	1.8835	-3.4846	0.0000	0.0061	-	-	-
TRINITY_DN30635_c2_g1_i1	1.8800	0.3746	0.0002	0.0495	REPAT30	2.00E-63	*Spodoptera* sp.
TRINITY_DN32840_c2_g1_i2	1.8271	-0.6562	0.0001	0.0248	-	-	-
TRINITY_DN39967_c1_g1_i5	1.7884	2.2680	0.0001	0.0314	Cytoplasmic dynein 1 intermediate chain isoform X8	0	*Amyelois transitella*
TRINITY_DN39493_c0_g3_i1	1.7112	-0.6333	0.0002	0.0393	Rho GTPase-activating protein 190-like	6.00E-44	*Plutella xylostella*
TRINITY_DN38296_c0_g1_i4	1.5741	0.0926	0.0001	0.0207	Uncharacterized protein	1.00E-103	*Danaus plexippus*
TRINITY_DN37877_c0_g1_i22	1.5721	-4.2336	0.2917	0.6661	-	-	-
TRINITY_DN39931_c0_g1_i9	1.5383	1.9613	0.0002	0.0415	Uncharacterized protein LOC101741686	0	*Bombyx mori*
TRINITY_DN37435_c0_g1_i4	1.3806	4.7764	0.0001	0.0298	Casein kinase I isoform gamma-3	0	*Pongo abelii*
TRINITY_DN33003_c3_g1_i2	1.2936	5.2805	0.0002	0.0438	-	-	-


**Table 3:** List of downregulated (log_2_-transformed fold change) transcripts recovered from brain tissue RNA extractions in bat call-exposed *Spodoptera frugiperda* adult male moths relative to controls, including the most significant (e-value < 1e-5) BLASTX protein annotation from the UniRef90 database and the organism from which the annotation is derived.

Transcript ID	Log_2_ fold change	Ave. expression	*P*-value	FDR-adjusted *P*-value	Top UniRef90 BLASTX Hit	E-value	Organism
TRINITY_DN22838_c0_g2_i1	-10.5503	0.8597	0.0000	0.0000	-	-	-
TRINITY_DN34268_c3_g1_i3	-10.3989	-0.6024	0.0000	0.0000	27 kDa hemolymph protein	5.00E-90	*Pararge aegeria*
TRINITY_DN40225_c4_g3_i5	-10.3616	-0.3944	0.0000	0.0001	-	-	-
TRINITY_DN34268_c4_g1_i1	-10.1455	-0.6638	0.0000	0.0000	-	-	-
TRINITY_DN25356_c0_g2_i1	-9.7357	0.4281	0.0000	0.0000	-	-	-
TRINITY_DN38145_c1_g1_i1	-9.4768	0.0648	0.0000	0.0000	-	-	-
TRINITY_DN38793_c0_g2_i2	-9.3882	-0.2339	0.0002	0.0498	Equilibrative nucleoside transporter	0	*Pararge aegeria*
TRINITY_DN36116_c4_g2_i6	-9.3085	-0.4714	0.0000	0.0008	-	-	-
TRINITY_DN38739_c1_g1_i5	-9.1023	-1.2984	0.0000	0.0000	Protein polybromo-1	0	*Papilio* sp.
TRINITY_DN33671_c2_g1_i1	-9.0696	-1.3407	0.0000	0.0000	-	-	-
TRINITY_DN32305_c5_g1_i3	-8.8475	-0.6495	0.0000	0.0042	-	-	-
TRINITY_DN35489_c0_g1_i6	-8.8298	-1.4900	0.0000	0.0003	-	-	-
TRINITY_DN24438_c0_g2_i1	-8.7905	1.2303	0.0000	0.0003	-	-	-
TRINITY_DN33318_c7_g1_i4	-8.7839	-1.2434	0.0000	0.0001	-	-	-
TRINITY_DN37153_c0_g3_i7	-8.6013	-1.5878	0.0000	0.0001	Voltage-dependent T-type calcium channel subunit alpha-1G	0	*Bombyx mori*
TRINITY_DN29335_c0_g1_i1	-8.5909	-1.4573	0.0000	0.0000	Uncharacterized protein LOC106129727	4.00E-36	*Amyelois transitella*
TRINITY_DN33003_c2_g1_i2	-8.5809	-0.4078	0.0000	0.0000	-	-	-
TRINITY_DN38739_c1_g1_i1	-8.5236	-1.5621	0.0000	0.0001	Protein polybromo-1	0	*Papilio* sp.
TRINITY_DN37612_c0_g1_i1	-8.4772	-1.6945	0.0001	0.0233	Peripheral-type benzodiazepine receptor isoform X1	4.00E-83	*Bombyx mori*
TRINITY_DN32142_c0_g2_i1	-8.4481	-1.5247	0.0000	0.0000	-	-	-
TRINITY_DN36507_c0_g1_i5	-8.4379	-0.1039	0.0001	0.0313	-	-	-
TRINITY_DN38898_c0_g1_i4	-8.3471	-0.2149	0.0000	0.0001	ADP ribosylation factor	1.00E-107	*Oryctes borbonicus*
TRINITY_DN37203_c0_g1_i2	-8.3042	-1.6302	0.0000	0.0000	Integrin beta pat-3	1.00E-100	*Danaus plexippus*
TRINITY_DN30280_c6_g1_i2	-8.1934	1.1569	0.0000	0.0047	-	-	-
TRINITY_DN35996_c6_g2_i7	-8.1751	-0.4905	0.0000	0.0002	Uncharacterized protein	2.00E-120	*Operophtera brumata*
TRINITY_DN33703_c0_g1_i10	-7.8473	-0.8351	0.0000	0.0000	FH1/FH2 domain-containing protein 3	0	*Bombyx mori*
TRINITY_DN32368_c2_g1_i1	-7.8392	-1.7751	0.0000	0.0000	-	-	-
TRINITY_DN33037_c7_g1_i1	-7.7746	-0.7471	0.0000	0.0013	-	-	-
TRINITY_DN32675_c1_g1_i2	-7.7181	-0.5445	0.0000	0.0061	-	-	-
TRINITY_DN35308_c0_g7_i2	-7.6312	-1.8407	0.0000	0.0000	Uncharacterized protein LOC106143546	0	*Amyelois transitella*
TRINITY_DN32480_c1_g1_i2	-7.6141	-1.7956	0.0000	0.0003	-	-	-
TRINITY_DN38739_c1_g1_i4	-7.5996	-1.8992	0.0000	0.0003	Protein polybromo-1	0	*Papilio* sp.
TRINITY_DN30400_c2_g1_i3	-7.5346	-1.9257	0.0000	0.0001	-	-	-
TRINITY_DN32071_c5_g2_i3	-7.5332	-0.9221	0.0000	0.0124	-	-	-
TRINITY_DN35282_c3_g2_i3	-7.5319	-1.5419	0.0001	0.0313	2-Methylene-furan-3-one reductase-like	0	*Bombyx mori*
TRINITY_DN39284_c17_g3_i1	-7.5016	-1.1912	0.0000	0.0001	Uncharacterized protein	4.00E-19	*Danaus plexippus*
TRINITY_DN36116_c4_g2_i3	-7.4942	-0.4979	0.0001	0.0332	-	-	-
TRINITY_DN37745_c1_g3_i1	-7.4529	-2.0874	0.0000	0.0000	-	-	-
TRINITY_DN38739_c1_g1_i12	-7.3904	-2.1667	0.0000	0.0014	Protein polybromo-1	0	*Papilio* sp.
TRINITY_DN32480_c1_g1_i1	-7.3853	-2.0826	0.0000	0.0000	-	-	-
TRINITY_DN35996_c6_g3_i1	-7.3769	-1.0123	0.0000	0.0009	-	-	-
TRINITY_DN37270_c2_g1_i3	-7.3114	-2.1964	0.0000	0.0001	-	-	-
TRINITY_DN37446_c0_g1_i5	-7.2694	-0.5738	0.0002	0.0435	-	-	-
TRINITY_DN34464_c1_g2_i7	-7.2006	-1.3360	0.0000	0.0008	Kv channel-interacting protein 4-like	2.00E-112	*Amyelois transitella*
TRINITY_DN34160_c1_g2_i1	-7.1791	-1.1829	0.0000	0.0000	DNA N^6^-methyl adenine demethylase-like isoform X1	2.00E-48	*Amyelois transitella*
TRINITY_DN35932_c1_g2_i2	-7.1349	-2.5094	0.0001	0.0308	Uncharacterized protein LOC106133073 isoform X1	8.00E-133	*Amyelois transitella*
TRINITY_DN32911_c0_g2_i1	-7.0475	-1.7120	0.0000	0.0001	-	-	-
TRINITY_DN38739_c1_g1_i14	-6.9385	-1.9898	0.0000	0.0000	Protein polybromo-1	0	*Papilio* sp.
TRINITY_DN39310_c1_g2_i4	-6.9376	-0.1062	0.0000	0.0024	Ankyrin repeat domain-containing protein 17-like	1.00E-121	*Papilio xuthus*
TRINITY_DN36781_c3_g1_i6	-6.9246	-1.4879	0.0002	0.0448	Cytochrome P450	0	*Spodoptera litura*
TRINITY_DN38815_c3_g4_i6	-6.8984	0.0770	0.0000	0.0000	-	-	-
TRINITY_DN32730_c0_g1_i3	-6.8032	-1.5119	0.0000	0.0000	Decaprenyl-diphosphate synthase subunit 2	0	*Amyelois transitella*
TRINITY_DN37877_c0_g1_i17	-6.7731	-1.4192	0.0000	0.0057	-	-	-
TRINITY_DN38024_c0_g2_i11	-6.7309	-1.2620	0.0000	0.0000	-	-	-
TRINITY_DN34405_c8_g4_i1	-6.7285	-1.5021	0.0000	0.0043	-	-	-
TRINITY_DN38296_c0_g1_i8	-6.7146	-1.4859	0.0000	0.0002	Uncharacterized protein	7.00E-104	*Danaus plexippus*
TRINITY_DN19414_c1_g1_i1	-6.6813	-1.5167	0.0000	0.0000	Glutamate synthase	3.00E-50	*Bombyx mori*
TRINITY_DN38328_c0_g1_i4	-6.6761	-2.2826	0.0000	0.0012	Uncharacterized protein	0	*Danaus plexippus*
TRINITY_DN38884_c1_g1_i14	-6.6106	-1.1688	0.0000	0.0022	-	-	-
TRINITY_DN35288_c0_g5_i3	-6.5728	-1.2283	0.0001	0.0234	-	-	-
TRINITY_DN37832_c2_g1_i1	-6.5413	-1.5530	0.0000	0.0000	-	-	-
TRINITY_DN38898_c0_g1_i3	-6.5141	-1.6907	0.0000	0.0008	-	-	-
TRINITY_DN37781_c1_g1_i6	-6.4907	-2.0293	0.0000	0.0010	Maltase 2-like isoform X1	4.00E-85	*Amyelois transitella*
TRINITY_DN32376_c4_g1_i4	-6.4609	-1.2338	0.0000	0.0009	-	-	-
TRINITY_DN33252_c1_g1_i3	-6.4412	-2.4937	0.0000	0.0000	-	-	-
TRINITY_DN36153_c0_g1_i3	-6.4325	-0.4561	0.0001	0.0254	Guanine nucleotide-binding protein-like 3 homolog	1.00E-92	*Papilio* sp.
TRINITY_DN30786_c0_g1_i4	-6.4324	-1.8255	0.0000	0.0000	-	-	-
TRINITY_DN34405_c8_g1_i1	-6.3667	-2.5342	0.0000	0.0002	-	-	-
TRINITY_DN38604_c4_g5_i2	-6.3565	-2.5853	0.0000	0.0000	-	-	-
TRINITY_DN34116_c1_g2_i1	-6.3322	-2.1255	0.0001	0.0167	Uncharacterized protein	2.00E-118	*Acyrthosiphon pisum*
TRINITY_DN19813_c0_g1_i1	-6.2812	-2.6099	0.0000	0.0000	-	-	-
TRINITY_DN19414_c0_g1_i1	-6.2801	-1.5813	0.0000	0.0000	Glutamate synthase NADH amyloplastic	6.00E-39	*Amyelois transitella*
TRINITY_DN32420_c1_g1_i2	-6.2359	-1.7600	0.0000	0.0001	-	-	-
TRINITY_DN34560_c0_g1_i3	-6.2113	-1.4242	0.0000	0.0009	Integrin beta	0	*Spodoptera frugiperda*
TRINITY_DN39620_c1_g1_i1	-6.1933	-2.6925	0.0000	0.0000	-	-	-
TRINITY_DN39051_c0_g2_i4	-6.1856	-1.5023	0.0000	0.0003	Uncharacterized protein	0	*Bombyx mori*
TRINITY_DN38145_c3_g1_i7	-6.1258	4.6107	0.0000	0.0007	Uncharacterized protein	4.00E-64	*Bombyx mori*
TRINITY_DN39075_c0_g1_i3	-6.1197	-2.0518	0.0000	0.0057	Uncharacterized protein	1.00E-82	*Bombyx mori*
TRINITY_DN37832_c3_g1_i1	-6.0722	-1.6664	0.0000	0.0036	-	-	-
TRINITY_DN32193_c2_g1_i4	-6.0557	-1.2068	0.0001	0.0284	Mitoferrin-1-like	9.00E-74	*Plutella xylostella*
TRINITY_DN32223_c5_g5_i2	-5.9885	-2.7475	0.0000	0.0000	-	-	-
TRINITY_DN39620_c1_g1_i3	-5.9855	-2.7377	0.0000	0.0000	-	-	-
TRINITY_DN13201_c0_g2_i1	-5.9681	-2.8018	0.0000	0.0000	Uncharacterized protein (fragment)	7.00E-06	*Piscirickettsia salmonis*
TRINITY_DN32859_c0_g1_i5	-5.9676	-2.3071	0.0000	0.0021	Putative pigeon protein	8.00E-97	*Danaus plexippus*
TRINITY_DN38145_c2_g1_i1	-5.9460	1.8448	0.0000	0.0001	-	-	-
TRINITY_DN40054_c3_g2_i4	-5.9348	-2.0780	0.0000	0.0001	WD repeat-containing protein 7 isoform X4	0	*Papilio* sp.
TRINITY_DN32169_c0_g1_i1	-5.9273	-2.4522	0.0000	0.0007	Muscle segmentation homeobox-like	2.00E-125	*Amyelois transitella*
TRINITY_DN28597_c2_g1_i2	-5.8573	-2.7815	0.0000	0.0001	-	-	-
TRINITY_DN38225_c0_g1_i1	-5.7856	6.9465	0.0001	0.0174	-	-	-
TRINITY_DN36707_c1_g1_i9	-5.7281	2.6878	0.0000	0.0001	Small conductance calcium-activated potassium channel protein	0	*Papilio polytes*
TRINITY_DN38163_c1_g2_i3	-5.7064	-0.9392	0.0000	0.0000	Catenin alpha	0	*Papilio polytes*
TRINITY_DN32901_c1_g5_i6	-5.6814	-2.8263	0.0000	0.0001	-	-	-
TRINITY_DN36307_c4_g1_i1	-5.6794	-2.9278	0.0000	0.0000	-	-	-
TRINITY_DN33558_c0_g2_i2	-5.6784	-1.7908	0.0000	0.0045	-	-	-
TRINITY_DN30964_c1_g2_i11	-5.6598	-2.8307	0.0000	0.0013	Ester hydrolase C11orf54 homolog	4.00E-136	*Amyelois transitella*
TRINITY_DN29565_c0_g1_i2	-5.6537	-2.9354	0.0000	0.0007	-	-	-
TRINITY_DN29467_c1_g1_i2	-5.5721	-0.9144	0.0001	0.0233	-	-	-
TRINITY_DN32901_c1_g5_i5	-5.5703	-2.7745	0.0000	0.0054	-	-	-
TRINITY_DN39896_c1_g2_i9	-5.5700	-2.0328	0.0000	0.0036	-	-	-
TRINITY_DN34685_c1_g1_i7	-5.4612	-3.0575	0.0000	0.0001	Laminin subunit alpha-1-like	2.00E-20	*Papilio machaon*
TRINITY_DN39620_c0_g1_i1	-5.4020	-3.0114	0.0000	0.0013	-	-	-
TRINITY_DN36528_c1_g3_i2	-5.3538	-0.7063	0.0001	0.0281	-	-	-
TRINITY_DN35630_c2_g1_i1	-5.3404	-2.3969	0.0002	0.0364	Retrovirus-related Pol polyprotein from type-2 retrotransposable element R2DM	0	*Ceratitis capitata*
TRINITY_DN39032_c0_g1_i9	-5.3317	-0.2183	0.0000	0.0016	Bromodomain-containing protein DDB_G0270170-like isoform X2	4.00E-133	*Papilio machaon*
TRINITY_DN38390_c0_g1_i4	-5.3096	-0.0925	0.0001	0.0209	Phosphatidylinositol 5-phosphate 4-kinase type-2 beta	0	*Ditrysia* sp.
TRINITY_DN37365_c2_g1_i1	-5.2580	-2.0441	0.0001	0.0248	Uncharacterized protein (Fragment)	1.00E-10	*Lottia gigantea*
TRINITY_DN32376_c4_g1_i2	-5.2177	-0.9262	0.0003	0.0586	-	-	-
TRINITY_DN39073_c3_g2_i13	-5.1049	-0.1081	0.0000	0.0141	ATP-citrate synthase	0	*Amyelois transitella*
TRINITY_DN37823_c2_g1_i8	-5.1015	-2.4653	0.0000	0.0001	Omega-amidase NIT2-A isoform X1	2.00E-145	*Amyelois transitella*
TRINITY_DN32098_c6_g2_i5	-5.0548	-1.6991	0.0001	0.0308	-	-	-
TRINITY_DN37877_c0_g1_i9	-4.9263	-1.0798	0.0003	0.0551	-	-	-
TRINITY_DN37877_c0_g1_i3	-4.9010	-1.0945	0.0001	0.0264	-	-	-
TRINITY_DN28575_c0_g1_i3	-4.8579	-2.8302	0.0000	0.0150	Solute carrier family 12 member 4 isoform X3	2.00E-22	*Papilio* sp.
TRINITY_DN28328_c0_g1_i3	-4.8107	-0.5775	0.0001	0.0237	-	-	-
TRINITY_DN29816_c0_g1_i2	-4.7990	4.0335	0.0000	0.0030	-	-	-
TRINITY_DN33031_c2_g2_i1	-4.7372	-2.7559	0.0000	0.0036	-	-	-
TRINITY_DN39545_c3_g1_i15	-4.6986	-2.8005	0.0001	0.0308	Endonuclease-reverse transcriptase	5.00E-21	*Bombyx mori*
TRINITY_DN31477_c1_g1_i4	-4.5937	-2.5111	0.0000	0.0149	Formin-like protein 15	2.00E-07	*Papilio machaon*
TRINITY_DN31395_c1_g1_i7	-4.5928	-3.1908	0.0003	0.0561	Arrestin homolog	0	*Obtectomera* sp.
TRINITY_DN34685_c1_g2_i2	-4.5483	-2.1143	0.0001	0.0309	Zinc finger MYM-type protein 1-like	6.00E-40	*Hydra vulgaris*
TRINITY_DN40097_c0_g1_i1	-4.4741	0.6703	0.0000	0.0009	c-Myc promoter-binding protein	0	*Homo sapiens*
TRINITY_DN38137_c0_g1_i4	-4.4587	-1.6514	0.0001	0.0360	Atrial natriuretic peptide-converting enzyme	0	*Bombyx mori*
TRINITY_DN36274_c0_g1_i2	-4.4428	-0.1042	0.0000	0.0001	Peptidyl-prolyl *cis–trans* isomerase FKBP65-like	5.00E-132	*Amyelois transitella*
TRINITY_DN37566_c0_g1_i2	-4.3099	-1.7781	0.0002	0.0444	-	-	-
TRINITY_DN35090_c0_g1_i3	-4.2946	-0.3012	0.0000	0.0043	Uncharacterized protein	3.00E-165	*Bombyx mori*
TRINITY_DN34211_c0_g1_i3	-4.2249	1.0640	0.0001	0.0178	Nuclear distribution protein NUDC	2.00E-161	*Biston betularia*
TRINITY_DN15012_c0_g2_i1	-4.1508	-1.2234	0.0001	0.0339	C-Cbl-associated protein isoform A	3.00E-10	*Operophtera brumata*
TRINITY_DN37270_c2_g1_i1	-4.1292	-3.6822	0.0002	0.0477	-	-	-
TRINITY_DN28005_c0_g1_i2	-4.1255	0.4174	0.0000	0.0017	39S ribosomal protein L34 mitochondrial	2.00E-36	*Papilio machaon*
TRINITY_DN28021_c0_g1_i1	-4.0897	-1.1177	0.0001	0.0233	Uncharacterized protein	3.00E-47	*Helobdella robusta*
TRINITY_DN40141_c1_g2_i1	-4.0186	-2.8432	0.0000	0.0010	Glutamate synthase (Fragment)	5.00E-38	*Pararge aegeria*
TRINITY_DN39099_c2_g1_i1	-3.9941	2.8840	0.0000	0.0061	-	-	-
TRINITY_DN36043_c0_g5_i1	-3.8734	-1.9678	0.0000	0.0091	-	-	-
TRINITY_DN37203_c0_g1_i3	-3.8576	3.1474	0.0000	0.0025	Integrin beta pat-3	8.00E-94	*Danaus plexippus*
TRINITY_DN37133_c4_g2_i5	-3.8209	-1.1337	0.0002	0.0402	-	-	-
TRINITY_DN31324_c0_g1_i3	-3.7809	-2.0764	0.0001	0.0207	Ubiquitin carboxyl-terminal hydrolase 34-like	2.00E-116	*Papilio machaon*
TRINITY_DN37153_c0_g3_i6	-3.6908	4.6006	0.0001	0.0207	Voltage-dependent T-type calcium channel subunit alpha-1G	0	*Bombyx mori*
TRINITY_DN39970_c7_g3_i1	-3.6002	-2.2744	0.0000	0.0036	-	-	-
TRINITY_DN36698_c2_g1_i3	-3.5166	-2.5650	0.0002	0.0369	Uncharacterized protein LOC106113347	3.00E-40	*Obtectomera* sp.
TRINITY_DN38059_c0_g1_i1	-3.4333	2.4478	0.0000	0.0053	Putative acetyltransferase ACT11	8.00E-102	*Spodoptera litura*
TRINITY_DN40211_c8_g13_i2	-3.3949	-2.6487	0.0000	0.0012	Uncharacterized protein	1.00E-17	*Piscirickettsia salmonis*
TRINITY_DN31644_c0_g1_i4	-3.2356	-1.6085	0.0000	0.0117	-	-	-
TRINITY_DN34933_c1_g1_i6	-3.1838	-1.7262	0.0000	0.0028	Collagen alpha-1(XXV) chain-like isoform X8	6.00E-69	*Bombyx mori*
TRINITY_DN39085_c0_g1_i6	-3.1011	4.5699	0.0000	0.0034	Uncharacterized protein LOC101738244	3.00E-153	*Bombyx mori*
TRINITY_DN33252_c1_g1_i1	-3.0698	2.3138	0.0000	0.0045	-	-	-
TRINITY_DN35322_c1_g2_i2	-2.9511	-1.3865	0.0003	0.0550	Putative zinc finger protein 91 (Fragment)	2.00E-92	*Operophtera brumata*
TRINITY_DN30567_c6_g2_i1	-2.9059	-1.5217	0.0000	0.0093	-	-	-
TRINITY_DN34764_c2_g2_i9	-2.8990	-2.6731	0.0001	0.0237	-	-	-
TRINITY_DN39515_c0_g1_i5	-2.7516	1.2549	0.0001	0.0286	Lachesin-like	0	*Bombyx mori*
TRINITY_DN33240_c0_g1_i6	-2.7220	-3.2631	0.0000	0.0017	Uncharacterized protein	2.00E-120	*Bombyx mori*
TRINITY_DN29565_c0_g2_i1	-2.6506	-1.7899	0.0000	0.0045	-	-	-
TRINITY_DN35544_c0_g3_i2	-2.6469	-0.4176	0.0000	0.0098	UPF0528 protein CG10038	6.00E-55	*Amyelois transitella*
TRINITY_DN38353_c3_g1_i8	-2.6247	-3.0481	0.0001	0.0232	-	-	-
TRINITY_DN38108_c0_g2_i6	-2.6245	2.6610	0.0000	0.0100	Uncharacterized protein LOC106136039	2.00E-152	*Amyelois transitella*
TRINITY_DN31477_c2_g1_i3	-2.5817	-0.6315	0.0000	0.0034	-	-	-
TRINITY_DN30567_c9_g1_i1	-2.5463	-1.0038	0.0000	0.0030	-	-	-
TRINITY_DN39985_c0_g1_i3	-2.5356	5.1760	0.0000	0.0027	Histone-lysine *N*-methyltransferase ash1	0	*Papilio* sp.
TRINITY_DN31213_c0_g1_i2	-2.4078	0.6190	0.0002	0.0472	GDNF-inducible zinc finger protein 1-like	9.00E-157	*Papilio* sp.
TRINITY_DN38047_c1_g1_i5	-2.2382	1.4642	0.0000	0.0068	Uncharacterized protein	6.00E-112	*Obtectomera* sp.
TRINITY_DN37035_c0_g10_i2	-2.1924	3.4328	0.0001	0.0339	-	-	-
TRINITY_DN37004_c7_g1_i3	-2.1562	-2.1811	0.0000	0.0133	-	-	-
TRINITY_DN39427_c3_g1_i2	-2.0552	-0.3606	0.0002	0.0488	Zinc finger protein 62 homolog isoform X2	2.00E-81	*Amyelois transitella*
TRINITY_DN31830_c4_g3_i2	-1.7960	-3.7434	0.0000	0.0043	Mucin-2-like	1.00E-59	*Amyelois transitella*
TRINITY_DN39449_c0_g1_i14	-1.7253	-3.5318	0.0001	0.0207	Uncharacterized protein	0	*Obtectomera* sp.
TRINITY_DN36559_c0_g2_i7	-1.7199	-0.8964	0.0001	0.0327	Uncharacterized protein	2.00E-44	*Danaus plexippus*
TRINITY_DN38544_c0_g3_i1	-1.7008	-1.8335	0.0002	0.0488	-	-	-
TRINITY_DN38707_c1_g2_i9	-1.6822	2.9542	0.0001	0.0237	Cytochrome P450 9A58	1.00E-166	*Spodoptera frugiperda*
TRINITY_DN37901_c0_g1_i4	-1.6242	1.0172	0.0001	0.0207	Uncharacterized protein LOC106132143	0	*Amyelois transitella*
TRINITY_DN37610_c5_g1_i3	-1.6189	0.6339	0.0001	0.0332	Uncharacterized protein LOC105397907	4.00E-92	*Plutella xylostella*
TRINITY_DN37496_c0_g2_i3	-1.5968	1.2808	0.0001	0.0178	Uncharacterized protein	5.00E-97	*Danaus plexippus*
TRINITY_DN32710_c0_g1_i1	-1.5769	2.2427	0.0001	0.0264	Uncharacterized protein	0	*Danaus plexippus*
TRINITY_DN39385_c2_g1_i5	-1.5003	5.7753	0.0001	0.0202	-	-	-
TRINITY_DN22676_c0_g1_i1	-1.4828	-0.6327	0.0001	0.0251	-	-	-


#### Upregulated Genes

Among the top 10 most highly upregulated genes were a X-linked retinitis pigmentosa GTPase regulator (RPGR) homolog and a mitochondrial calcium uniporter protein, though seven genes were unannotated, including the most highly upregulated transcript, with the remaining transcripts annotated by uncharacterized proteins. Genes also had highly variable absolute log_2_-transformed fold changes (log_2_FC) ranging from 1.29 to 11.45. Additional upregulated genes of interest include the regulatory-associated protein of TOR, axin, inositol 1,4 5-triphosphate 5-phosphatase, Hsp 67B2-like isoform X2, glutathione (GSH) *S*-transferase 2-like, and the rho GTPase-activating protein.

#### Downregulated Genes

The top 10 most downregulated genes included three with annotations, a 27 kDa hemolymph protein, an equilibrative nucleoside transporter, and protein polybromo-1 (Pb-1), while the remaining seven failed to be annotated. Again, absolute fold-change expression varied broadly (1.48–10.55 log_2_FC) though several other annotated and functionally relevant genes were downregulated. In particular, voltage-gated ion channels, DNA *N*^6^-methyl adenine (6mA) demethylase-like isoform, histone-lysine *N*-methyltransferase, phosphatidylinositol 5-phosphae 4-kinase, two different cytochrome P450s, glutamate synthase, integrin beta, mitoferrin-1, ankyrin repeat domain-containing protein 17, arrestin, and several zinc finger proteins.

### Gene Ontology Enrichment Analysis and KEGG Pathway Reconstruction

**FIGURE 2:**
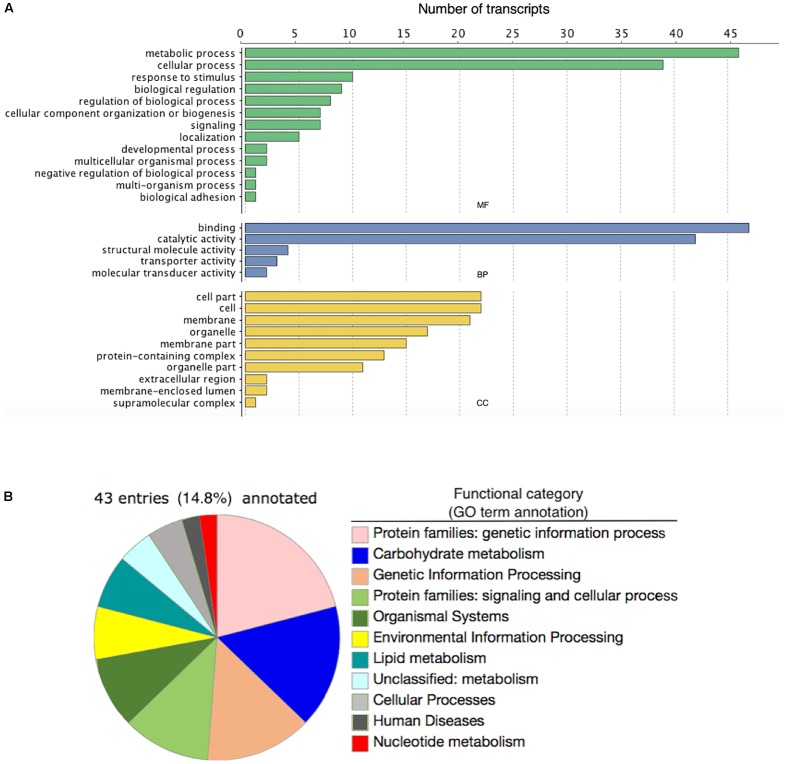
**(A)** The number of differentially expressed transcripts annotated with Gene Ontology terms corresponding to either a molecular function (MF; green bars), biological process (BP; blue bars), or cellular component (CC; yellow bars) in the brains of four adult male fall armyworm (*Spodoptera frugiperda*) moths exposed to recorded bat foraging and attack calls for 8 h. **(B)** Pie graph detailing the Kyoto Encyclopedia of Genes and Genomes (KEGG) Orthology annotations associated with differentially expressed transcripts in the male brains of adult fall armyworm moths exposed for 8 h to recorded bat foraging and attack calls.

Of the 146 DE annotated transcripts, 102 (69.8%) displayed GO term sequence identity (Figure 2A). GO term enrichment analysis identified 15 overrepresented and 0 underrepresented GO categories in our exposed samples (FDR-adjusted *p*-value < 0.05; Table 4). Six of these overrepresented GO terms pertained to glutamate metabolism, biosynthesis, and synthase activity, while dicarboxylic acid biosynthesis and metabolism corresponded to two terms, and oxidoreductase, aminoacylase, flavin mononucleotide binding, chromatin binding, and macromolecular complex binding corresponded to one term each. Notably, 14 of these 15 overrepresented GO terms annotated a downregulated transcript while only a single term pertained to an upregulated transcript. Of note is that the majority of transcripts mapping to significantly enriched GO terms occurred as very low or zero transcript count observations in the exposed relative to the control group. All transcripts mapping to chromatin binding-, glutamate-, integrin-, oxidoreductase-, and aminoacylase-related GO terms exhibited this pattern of “all-or-nothing” transcript expression. As the data included considerable noise, the prevalence of this pattern among the differentially expressed GO annotated transcripts may simply be due to these patterns being the only ones strong enough to discern statistically, though their functional relevance in stress physiology requires further investigation. Our BlastKOALA KEGG pathway reconstruction of the 290 DE transcripts recovered 43 (14.8%) with functional annotations, including 37 pertaining to cellular metabolism, six related to genetic information processing, nine that function in cellular signal transduction to environmental stimuli, five related to cell growth and death, two related to glutamatergic and GABAergic synapses, respectively, and one related to neurotrophin signaling in neurons specifically (Figure 2B).

**Table 4:** List of statistically over-represented (hypergeometric test, FDR-adj. *p* < 0.05) Gene Ontology (GO) term annotations associated with the 290 differentially expressed (DE) transcripts identified after frequent, prolonged bat-ultrasound exposure in brain tissue of adult male *Spodoptera frugiperda* moths.

GO category	GO ID	Description	DE cluster frequency	GO-annotated transcriptome frequency	FDR-adjusted *P-*value
Biological process	6536	Glutamate metabolic process	3/102 (2.9%)	10/40511 (0.1%)	1.84E-06
	6537	Glutamate biosynthetic process	3/102 (2.9%)	10/40511 (0.1%)	1.84E-06
	43650	Dicarboxylic acid biosynthetic process	3/102 (2.9%)	21/40511 (0.1%)	1.99E-05
	7229	Integrin-mediated signaling pathway	4/102 (3.9%)	89/40511 (0.1%)	7.85E-05
	43648	Dicarboxylic acid metabolic process	3/102 (2.9%)	53/40511 (0.1%)	3.31E-04
	9084	Glutamine family amino acid biosynthetic process	3/102 (2.9%)	64/40511 (0.1%)	5.77E-04
Molecular function	3682	Chromatin binding	7/102 (6.8%)	77/40511 (0.1%)	1.08E-09
	44877	Macromolecular complex binding	7/102 (6.8%)	135/40511 (0.1%)	5.54E-08
	15930	Glutamate synthase activity	3/102 (2.9%)	5/40511 (0.1%)	1.54E-07
	45181	Glutamate synthase activity, NAD(P)H as acceptor	2/102 (1.9%)	4/40511 (0.1%)	3.75E-05
	16040	Glutamate synthase (NADH) activity	2/102 (1.9%)	4/40511 (0.1%)	3.75E-05
	10181	FMN binding	3/102 (2.9%)	49/40511 (0.1%)	2.62E-04
	16639	Oxidoreductase activity, acting on the CH-NH2 group of donors, NAD or NADP as acceptor	2/102 (1.9%)	11/40511 (0.1%)	3.40E-04
	16638	Oxidoreductase activity, acting on the CH-NH2 group of donors	3/102 (2.9%)	55/40511 (0.1%)	3.70E-04
	4046	Aminoacylase activity	2/102 (1.9%)	12/40511 (0.1%)	4.08E-04


## Discussion

### A Comparison of Predator-Induced Gene Expression Responses in Other Animals

Our results build on a growing body of literature detailing auditory sensory mode and predator-induced shifts in gene expression in vertebrates and invertebrates (Nanda et al., 2008; Leder et al., 2009; Preisser, 2009; Sheriff and Thaler, 2014; Takahashi, 2014; Harris and Carr, 2016; Adamo, 2017a,b). Several studies have focused on describing the gene expression dynamics of large-scale predator-induced morphological changes that occur in organisms displaying predation-related polyphenisms, including multiple species of *Daphnia* (Schwarzenberger et al., 2009; Spanier et al., 2010; Rozenberg et al., 2015) and the Hokkaido salamander (*Hynobius retardatus*; Matsunami et al., 2015). Less striking predator-induced changes also have been studied in diverse taxa, including stickleback fish (Sanogo et al., 2011) and an intertidal snail (Chu et al., 2014). Exposure to auditory cues of aerial hawking bats for 8 h resulted in significant transcriptomic responses, as evidenced by the wide-ranging fold-changes (log_2_FC) in transcript expression reported here. In the brains of predator-stressed sticklebacks, low-to-moderate fold-changes ranged from 2 to 6 (log_2_FC; Sanogo et al., 2011), while predator-induced polyphenic *Daphnia* displayed changes ranging from 2 to 10 (log_2_FC; Rozenberg et al., 2015). Furthermore, the number of DE transcripts found here is comparable to that found in other RNA-seq studies on predator-induced gene expression among invertebrates. For instance, *Daphnia pulex* exposed to kairomones of predatory phantom midge (*Chaoborus*) larvae displayed 256 DE transcripts (Rozenberg et al., 2015), while only three transcripts were differentially regulated in the intertidal snail *Nucella lapillus* when exposed to seawater that flowed first through a chamber holding a predatory crab (*Carcinus maenas*) feeding on *N. lapillus* (Chu et al., 2014). Further, the number of DE transcripts from brain tissue after predator exposure can vary strongly based on predator identity, as shown by Matsunami et al. (2015) who found that Hokkaido salamander larvae exposed to predatory dragonfly naiads displayed 605 DE transcripts, while only 103 DE transcripts were found after exposure to predatory tadpoles. One primary difference between past studies of predator-induced transcriptional changes that must be considered when interpreting the results presented here is the time scale at which cues of predation are presented. In the case of predator-induced polyphenisms, exposure length depends highly on organism life history but ranges generally from a few to several days. Though our study assesses the effects of prolonged, frequent exposure to an auditory cue of predation over a single night, it should be noted that this time scale is much shorter than used in most other studies of predator-induced transcription. Clearly, the degree to which prey respond transcriptionally to cues of predation risk can vary broadly across taxa and no clear pattern has yet emerged. However, the ubiquity with which metazoan life responds transcriptionally to these cues of predation begs the detailed description of these gene pathways, their relevance to physiology and life history, and their evolution throughout the tree of life.

### Functional Relevance of Differentially Regulated Genes

Furthermore, our results indicate a broad range of functional annotations related to our DE transcripts. For instance, upregulated transcripts coded for proteins related to cellular signaling, Hsp synthesis, antioxidant metabolism, mitochondrial metabolism, oxidoreductase activity, glutamate synthesis, ionotropic receptor activity, gene regulation, ion transport, and cilium assembly. Downregulated transcript annotations also displayed a large degree of functional variability relating to G-coupled protein signaling, cytochrome P450 activity, chromatin-mediated gene regulation, integrin signaling, glutamate biosynthesis, and voltage-dependent ion channels, among others. Several notable transcript upregulations corresponded to unexpected protein annotations, including a mitochondrial calcium uniporter protein (log_2_FC = 9.66), an RPGR homolog (log_2_FC = 9.86), mutant cadherin (log_2_FC = 5.60), mitochondrial choline dehydrogenase (log_2_FC = 6.78), and acyl-coenzyme A synthetase short-chain family member 3 (log_2_FC = 6.22). The mitochondrial calcium uniporter protein acts as a transmembrane transporter for uptake of calcium ions into mitochondria for use during respiration (Marchi and Pinton, 2014) after these ions are mobilized from intracellular stores by inositol triphosphate. Notably, another significantly upregulated gene among exposed individuals was type 2 inositol 1,4,5-triphosphate 5-phosphatase (log_2_FC = 6.06). In humans, this phosphatase hydrolyzes inositol triphosphate and functions as a signal-terminating enzyme, preventing further calcium release (Ross et al., 1991; Contreras et al., 2010).

The second most upregulated transcript codes for a RPGR homolog, a protein usually associated with cilia development in the photoreceptors of vertebrate eyes (Gakovic et al., 2011), although it localizes to other tissues and cell types as well (Khanna et al., 2005). We suggest that RPGR upregulation may be related to increased cilia development and neuronal connections but since its expression has not been studied in insect eyes or other tissues, further conclusions about the function of this protein under predator-stressed conditions in *S. frugiperda* cannot be made. Because *S. frugiperda* brains were excised without compromising pigment-storing ommatidial cells, the RPGR expression pattern observed here likely is intrinsic to brain tissue and may be related to neural tissues extending from innervations of the eye. Notably, the entire suite of phototransduction proteins found in the *Drosophila* visual system is also found to act in the fly’s auditory transduction system, with visual rhodopsins serving mechanical transduction and amplification roles in auditory neurons of the Johnston’s organ (Pumphrey, 1940).

Another upregulated transcript that may be related to neuronal development encoded a mutant cadherin protein found in humans. Cadherins are calcium-dependent cell-cell adhesion proteins that are integral in nearly every step of neural development in larval *Drosophila* (Fung et al., 2009), have been implicated in guiding new neuron development contributing to neural plasticity (Edsbagge et al., 2004), and are even involved in hair bundle development in vertebrate ears (Hirano and Takeichi, 2012). As expression of cadherins is usually repressed and localized only to synaptic areas in mature brain tissues (Hirano and Takeichi, 2012), the fact that it is highly upregulated in predator-cue exposed *S. frugiperda* coupled with RPGR upregulation suggests that neural plasticity and development of new neural connections upon exposure to novel environmental cues may play key roles in functionally responding to auditory predator cues.

Several strongly downregulated transcripts also mapped to unexpected protein annotations, including a 27 kDa hemolymph protein (log_2_FC = -10.40), DNA 6mA demethylase-like isoform X1 (log_2_FC = -7.18), decaprenyl-diphosphate synthase subunit 2 (DDSS2; log_2_FC = -6.80), FH1/FH2 domain-containing protein 3 (FHOD3; log_2_FC = -7.85), and Pb-1 (isoform log_2_FC = -9.10, -8.52, -7.60, -7.39, -6.94, 8.54). The 27 kDa hemolymph protein family consists of proteins found in diverse insect taxa but their function remains unknown. DNA 6mA demethylase is another enzyme correlated with a highly downregulated transcript. Methylation of 6mA has been studied primarily in prokaryotes, where it serves as the primary mechanism for epigenetic signaling via DNA methylation—as opposed to the primary mechanism found in eukaryotes, 5-methylcytosine methylation (Vanyushin et al., 1968). Demethylases associated with 6mA and 5-methylcytosine serve to remove methyl groups from DNA and RNA, affecting the transcription and translation of affected nucleic acid chains. In plants and vertebrates, 6mA methylation both increases and decreases transcription factor binding (Luo et al., 2015), while in *Drosophila melanogaster* loss of a putative 6mA demethylase resulted in increased transposon expression (Zhang et al., 2013). Notably, a transcript annotated with histone-lysine *N*-methyltransferase (log_2_FC = -2.54) and five transcript isoforms annotated with Pb-1 were downregulated after predator-cue exposure, although another Pb-1 isoform was also upregulated. These proteins are involved in histone H3 remodeling and binding, respectively (Chandrasekaran and Thompson, 2007; An et al., 2011). Although the functional significance of these downregulated genes in the brain of predator-exposed *S. frugiperda* is unclear, epigenetic mechanisms appear to be induced in some manner.

The enzyme DDSS2 catalyzes a reaction to supply decaprenyl diphosphate for use in ubiquinone-10 biosynthesis. Ubiquinone-10 is concentrated in mitochondria, where it acts as a component of the electron transport chain during aerobic cellular respiration (Ernster and Dallner, 1995), although it also is found in many diverse organelles at lower concentrations. In this context, ubiquinone-10 acts as an electron transport enzyme moving electrons from enzyme complexes I and II to III in the electron transport chain, a function only it and vitamin K_2_ are able to perform (Bhalerao and Clandinin, 2012). Ubiquinone-10 also serves as an antioxidant due to its weak electron affinity when reduced. In this state, electrons are held so loosely that the molecule readily gives up electrons to oxidized substrates. For instance, within mitochondria, ubiquinone-10 prevents the oxidation of DNA nucleotides during interactions between peroxidase and DNA-bound metal ions (López et al., 2010; Miyamae et al., 2013). Although the down-regulation of DDSS2 does not directly imply that lower levels of ubiquinone-10 were present in predator-cue exposed *S. frugiperda*, further studies should examine ubiquinone-10 responses to predator exposure. With knowledge of the increased mitochondrial metabolic activity suggested by several upregulated transcripts discussed previously, it is surprising that DDSS2 is downregulated, as a greater need for electron transport substrates and antioxidants with enhanced energy production might be expected. Clearly, there is still much to learn in elucidating the role of DDSS2, and mitochondrial metabolism in general, in the context of predator-induced stress responses.

Formin homology 1/formin homology 2 domain-containing protein 3 (FHOD3), another protein that mapped to a highly downregulated transcript in the predator-exposed *S. frugiperda* brain, acts as an actin regulator with a scaffolding function and has been found, in humans, to affect organogenesis, tissue homeostasis, and cancer-cell invasion (Katoh and Katoh, 2004). Actin, a protein that forms microfilaments and constitutes the actin cytoskeleton in all eukaryotic cells, plays a key role in cellular locomotion and shape (Lodish et al., 2000). FHOD family proteins are thought to bind to the growing barbed-end of actin polymers and serve both to deliver new actin monomers and promote actin polymerization, effectively mediating the growth of the actin cytoskeleton (Bechtold et al., 2014). FHOD family proteins are regulated by rho-GTPases, a member of which was downregulated after predator-exposure. Furthermore, actin-binding Lin11, Isl-1, Mec-3 protein 3 and alpha catenin were also down-regulated and act as a scaffold protein (Barrientos et al., 2007) and a cellular linking protein between cadherins and actin-containing filaments (Geoffrey and Robert, 2000; Drees et al., 2005; Yamada et al., 2005), respectively. Considering that a transcript encoding a mutant cadherin was upregulated in predator-exposed brains as well, these patterns suggest that the actin cytoskeleton is affected by predator-exposure and that changes in cellular morphology and motility may be involved.

### Overrepresented Gene Ontology Terms and KEGG Pathway Reconstruction in Predator-Stressed Brain Tissue

Relative to the list of DE transcript annotations in this study, the overrepresented GO terms enriched in the brains of *S. frugiperda* after predator exposure were generally restricted to three biochemical pathways: (1) chromatin and macromolecule binding, (2) glutamate synthesis and metabolism, and (3) aminoacylase activity, although terms related to oxidoreductase activity, flavin mononucleotide binding, and integrin signaling also were overrepresented. To the best of our knowledge, these GO terms have not been implicated in any other study of predator-induced transcription. The small set of GO-annotated DE transcripts identified here limit the statistical detection of subtly over- and under-represented terms; regardless, we found 15 GO terms to be highly significantly overrepresented in our set of annotated DE transcripts relative to the frequency at which these terms were found in our GO-annotated transcriptome (*p* < 0.0004). Chromatin binding (*p* < 0.0000) and macromolecular complex binding (*p* < 0.0000) were the most highly overrepresented GO terms identified both with 7 out of 102 GO-annotated DE transcripts mapped to these terms. The binding of cellular proteins to chromatin can elicit varied cellular responses, such as transcriptional regulation, DNA replication, and chromatin remodeling (Ricke and Bielinsky, 2005). Considering that transcripts mapping to ash1 and Pb-1 protein annotations were also differentially regulated, the presence of these GO terms again implies that epigenetic modifications seem to be induced upon exposure to predator cues.

The set of GO terms pertaining to glutamate synthesis and metabolism included glutamate synthase activity, as well as glutamate biosynthesis and metabolism, glutamine family amino acid biosynthesis, and dicarboxylic acid biosynthesis and metabolism. Glutamate, an amino acid anion derived from its dicarboxylic state, glutamic acid, is used during protein synthesis, but is the most abundant excitatory neurotransmitter in the vertebrate brain (Locatelli, 2005). Although acetylcholine is the primary excitatory neurotransmitter in the insect nervous system (Wnuk et al., 2014), glutamate also plays an excitatory role (Leboulle, 2012) as glutamate immunoreactivity (Sinakevitch et al., 2001) and glutamate-induced ion currents (Cayre et al., 1999) have been observed in insect neurons. Intriguingly, application of glutamate to the mushroom body brain regions of the honeybee, *Apis mellifera*, facilitates glutamatergic neurotransmission and olfactory learning (Locatelli, 2005), and glutamate-mediated neurotransmission has also been implicated in the visual and tactile (Liang et al., 2012) sensory systems. Notably, one of the strongly upregulated (log_2_FC = 5.70) transcripts we found mapped to a fragment of the ionotropic receptor 75d (IR75d). Benton et al. (2009) found that IR75d and 69 other IR-family proteins carry ionotropic glutamate receptor-like amino acid positions and surmised that IRs may similarly act in chemosensory neuron signaling. Although 21 of these 69 novel IRs showed transcriptional responses to chemical signals in the *Drosophila* antenna, including IR75d, the remaining 46 displayed no chemosensory-related expression (Benton et al., 2009). Further, the presence of different IR subtypes on a given neuron also influences synaptogenesis, synaptic activity, and experience-dependent neural plasticity in *Drosophila* (Thomas and Sigrist, 2012). Knowing that biochemical pathways pertaining to glutamate production were altered in the brains of predator-cue exposed *S. frugiperda* coupled with evidence that IR75d was upregulated post-exposure, we suggest that IR75d and its relatives may be involved in the development and function of auditory mechanosensory neurons.

Manual analysis of the KEGG pathway reconstruction of DE transcripts revealed a variety of interconnected neuron-specific metabolic and signaling cascades that were affected by bat ultrasound exposure, including the mechanistic target of rapamycin (mTOR)/Akt, MAPK, Wnt, prolactin, Hippo, and calcium signaling systems, and associated regulatory responses, such as p53, renin-angiotensin, and NF-κB transcript expression. Notably, recent research on the conserved function of these biochemical pathways in the nervous systems of metazoan taxa across phyla describes the function and biological relevance of these pathways on an organismal scale (Mattson and Camandola, 2001; Lilienbaum and Israe, 2003; Pan, 2007; Lau and Bading, 2009; Tedeschi and Di Giovanni, 2009; Brown et al., 2012; Lin et al., 2012; Graber et al., 2013; Flentke et al., 2014; Mao et al., 2014; Patil et al., 2014; Layden et al., 2016; Guo et al., 2017; Haspula and Clark, 2018). For instance, synaptic glutamate (Sinakevitch et al., 2010; Thomas and Sigrist, 2012; Li et al., 2016), mTOR/Akt (Guo et al., 2017), intracellular calcium (Kaltschmidt et al., 2005; Lau and Bading, 2009), and prolactin signaling (Brown et al., 2012; Belugin et al., 2013), followed by differential p53 and NF-κB transcription (Kaltschmidt et al., 2005; Lau and Bading, 2009) are each implicated in the apoptotic and synaptic-activity mediated induction of neural plasticity, learning, and memory from diverse taxa spanning arthropods to chordates. Clearly, much work remains to divulge how the vast evolutionary divergences inherent between the conserved physiological cellular signaling and gene networks of most, if not all, metazoan taxa correlate with lineage and ecology-specific organismal responses to diverse stressors, including predation risk.

## Conclusion and Future Directions

This study demonstrates that exposure to ecologically relevant auditory cues of predation risk in *S. frugiperda* results in varied but strong patterns of up- and down-regulation of a broad range of protein products within the moth brain. The most strongly up- and down-regulated transcripts found in this study correspond to many cellular functions which include mitochondrial metabolism, glutamate synthesis and metabolism, actin cytoskeleton morphology and motion, axon guidance, neural structure, and epigenetic modifications. This is a promising first step in developing a model for the transcriptional impacts of frequent and repeated exposure to bat predation cues in *S. frugiperda*, which may represent acute and chronic responses of cells to predator-induced stress. Several novel predator-cue induced transcriptional pathways are implicated in these results and present promising opportunities for future research. These broad predator-induced transcriptional responses are characteristic of those found in previous studies, such as in predator-stressed stickleback fish (Sanogo et al., 2011), *Daphnia* (Rozenberg et al., 2015), and the Hokkaido salamander (Matsunami et al., 2015). Contrary to our expectations, there is little overlap between previously reported responses to predator-induced stress, such as neuropeptide production and increased antioxidant activity, and the novel predator-induced functional annotations reported here. However, mitoferrin, a solute carrier responsible for iron uptake by red blood cells in vertebrates, was significantly upregulated in the brains of stickleback fish repeatedly exposed to a chemical cue of predation (Sanogo et al., 2011), although it was downregulated (log_2_FC = -6.06) in *S. frugiperda* post-exposure. In insects, the function of mitoferrin is less well understood, though *D. melanogaster* with mitoferrin mutations experienced problems with spermatogenesis and development to adulthood (Metzendorf and Lind, 2010). Apart from this similarity, the novel transcriptional responses to predation in *S. frugiperda* observed here may be specialized to auditory perception or found only in Lepidoptera. Furthermore, although efforts were made to avoid auditory habituation in this study, expression profiles described here bear similarities to past studies of bird-song habituation in the brains of zebra finches (*Taeniopygia guttata*), with both resulting in the downregulation of genes pertaining to cytoskeletal dynamics and mitochondrial metabolism (Dong et al., 2009).

One primary limitation of our study is a lack of time-series expression data that would have bolstered our ability to infer the functional relevance of specific transcripts for both short- and long-term physiological acclimations to auditory cues of predation. Further work, such as comparing expression profiles through time and between frequent and infrequent cue exposures, would aid in parsing the effects due to neural habituation/auditory stimulation, *per se*, and those related specifically to predator cue exposure. Specifically, producing a detailed time-course transcriptional profile of tissue-specific prey physiology beginning after the first moments of predator-cue exposure and proceeding over the course of hours to days in cue-exposed *S. frugiperda* or other predator–prey systems would provide comparative insights into the temporal dynamics of stress-induced transcription during acute relative to prolonged exposure to predation risk. Another limitation of this study is a lack of transcript validation via quantitative reverse-transcriptase polymerase chain reaction assays, yet we argue the novelty of the system and the foundational datasets we have produced that can inform future hypotheses warrant their use by the scientific community. Another confounding factor that may have contributed to the relatively noisy patterns of expression in exposed *S. frugiperda* brains shown here is the type of auditory stimulus we used. For instance, by using three bat calls from three different species, we have endeavored to replicate an ecologically relevant cue of predation risk, yet the nightly soundscape a moth is exposed to *in situ* varies hour-to-hour and night-to-night in sound intensity, conspecific and interspecific composition, and many other attributes that we did not incorporate into our experiments. We encourage future investigators to develop high quality, ultrasonic soundscape recordings in relevant field settings ahead of time, when possible, and replicate these via nightly broadcasts of each night’s recording. In conclusion, as more diverse, annotated insect genomes become available and the function of more genes are elucidated by experimental and comparative evidence, studies that assess the physiological effects of prolonged predation risk on prey across the tree of life will continue to divulge remarkably conserved patterns of stress-induced molecular mechanisms between lineages.

## Author Contributions

SC conducted the experiments and developed this report. ST contributed to the conceptual development, logistical support, and proofreading of this work.

## Conflict of Interest Statement

The authors declare that the research was conducted in the absence of any commercial or financial relationships that could be construed as a potential conflict of interest.

## Footnotes

1 https://sourceforge.net/projects/cdbfasta/

2 https://www.ncbi.nlm.nih.gov/bioproject/PRJNA318819

3 https://github.com/cinel1/FallArmyworm.git
